# The expression profile of miR-23b is not altered in peripheral blood mononuclear cells of patients with idiopathic inflammatory myopathies

**DOI:** 10.12688/f1000research.2-223.v1

**Published:** 2013-10-23

**Authors:** Martina Remakova, Tana Svitalkova, Marek Skoda, Jiri Vencovsky, Peter Novota

**Affiliations:** 1 Department of Rheumatology, First Faculty of Medicine, Charles University in Prague and Rheumatology Institute, Prague, Czech Republic; 2 Department of Experimental and Clinical Rheumatology, Institute of Rheumatology, Prague, Czech Republic

## Abstract

Idiopathic inflammatory myopathies (IIM) belong to a group of autoimmune disorders, primarily characterized by chronic inflammation of human skeletal muscle tissue. The etiology of these diseases is unknown, however, genetic predisposition plays a significant role in disease onset. Beside the known genetic risk located in the MHC complex, the epigenetic modifications including changes in miRNAs expression profiles have been recently implicated recently in many autoimmune diseases. Micro RNA molecules are involved in many physiological processes, including the regulation of the immune response.

In our study we have focused on the miR-23b, as it represents a novel promising autoimmunity regulator molecule. Downregulation of miR-23b was recently described in patients with rheumatoid arthritis and systemic lupus erythematosus. We have measured the expression miR-23b peripheral blood mononuclear cells of patients with dermatomyositis and polymyositis. No meaningful difference was found in comparison with healthy controls.

## Introduction and aims

Idiopathic inflammatory myopathies (IIM) are chronic autoimmune disorders characterized by skeletal muscle weakness, inflammatory immune cells in muscle tissues and frequent presence of serum autoantibodies. IIM can be divided into several groups: dermatomyositis (DM), polymyositis (PM), inclusion body myositis (IBM), and immune mediated necrotizing myopathy (NM). The etiology of IIM remains unclear; however current findings indicate that epigenetic mechanisms may contribute to the disease pathogenesis ^1, 2^.

MicroRNA molecules are part of the epigenetic regulatory network. They act as negative regulators of gene expression and participate in the regulation of key biological processes such as cell growth, differentiation, proliferation or apoptosis ^3– 6^. Recent findings highlight the crucial importance of miRNAs in development, homeostasis and function of innate and adaptive immunity ^7– 10^. Aberrant expression patterns have been documented in a broad range of diseases including autoimmune disorders ^11^.

In our study we have focused on the miR-23b type of microRNA, as it represents a novel promising autoimmunity regulator molecule with considerable therapeutic potential ^12^. Recent research has revealed downregulation of this miRNA in resident cells present in inflammatory lesions of patients with rheumatoid arthritis (RA) and systemic lupus erythematosus (SLE) and its role during the pathogenesis of autoimmune disease has been functionally characterized ^12^.

The aim of our study was to analyze differential expression of miR-23b and miR-23b* in peripheral blood mononuclear cells (PBMCs) of patients with PM/DM, and to examine potential involvement of these miRNA types in the pathogenesis of IIM.

## Material and methods

A cohort of 25 adult patients (age range 21–86 years, 7 males) suffering from idiopathic inflammatory myopathy and 22 healthy controls (age range 25–79 years, 5 males), both from the middle region of Czech Republic were analyzed. Diagnosis of PM or DM was determined according to the Bohan and Peter criteria ^13, 14^. In total 12 of patients suffered from PM, 13 patients were diagnosed as DM. All individuals involved in this study signed an informed consent. PBMCs of all individuals were obtained and purified by density gradient centrifugation on Ficoll in Leucosep tubes (Greiner Bio-One GmbH, Germany). 10 ml of peripheral blood of patients with myositis and healthy control subjects was collected into EDTA coated tubes under sterile conditions. The blood was added to a LeucoSep tube. The cell separation tubes were centrifuged (Jouan CR 3i; Jouan, St. Herblain, France) for 10 min at 2400 rpm without braking at room temperature. The cell suspension was collected, and the cells were washed three times in PBS (for 10 min at 1000 rpm), resuspended in 1 ml PBS and transferred into a 1,5 ml microcentrifuge tube. The microcentrifuge tube was centrifuged at 8000 rpm for 1 minute, supernatant was removed and the cell pellet was frozen using liquid nitrogen and stored at -80°C. The total RNA preparation from PBMCs was carried out using the conventional TRIZOL ^®^ reagent (Invitrogen, Carlsbad, USA) extraction procedure ^15^. Afterwards, the RNA was treated with RQ1 RNase free DNase (Promega, USA) for 20 min at 37°C in order to remove genomic DNA contamination, purified with phenol-chloroform-isoamylalcohol (25:24:1, PENTA, Czech Republic) reextraction and precipitated by 1 volume 2-propanol and 1/10 volume 300 mM sodium acetate (pH 4.8). Then, the RNA was washed twice with 70% ethanol and dissolved in RNase-free water (Life Technologies, Carlsbad, CA, USA). The quantity of total RNA was measured with a NanoDrop 2000 spectrophotometer (NanoDrop Technologies, USA). A microfluidic electrophoresis 2100 Bioanalyzer (Agilent Technologies, USA) was then used to quantify miRNA in absolute amounts [pg] and as a percentage of small RNA [%].

TaqMan gene specific miRNA assays were used to quantify the expression levels of mature miR-23b and miR-23b* (microRNA 000400, 002126) from Life Technologies (Carlsbad, CA, USA). Total RNA was reverse-transcribed by the TaqMan microRNA reverse transcription kit (Applied Biosystems) in a reaction mixture containing a miR-specific stem-loop reverse transcription (RT) primer. The quantification of mature miRNAs was performed using the TaqMan miRNA assay kit (Applied Biosystems) containing TaqMan primers in a universal PCR master mix without AmpErase UNG. 4 small nuclear RNAs - RNU44, RNU48, U47 and RNU6B (microRNA 001094, 001006, 001223, 001093, Applied Biosystems) were amplified as an internal control. qPCR was conducted at 95°C for 10 min, followed by 40 cycles of 95°C for 15 s and 60°C for 1 min. The expression level of miR-23b (miR-23b*) is shown as delta Ct. The delta Ct value was normalized to the mean value of four other miRNA molecules used in this experiment as housekeeping molecules (RNU44, RNU48, RNU6B, U47). This process was used to overcome the differences in cDNA quantity between different samples. All analyses were done in technical duplicates.

Statistical significance was determined by the Student's t-test using Microsoft Excel ^®^2007. A value of P<0.05 was considered to be statistically significant.

The whole study, including the laboratory work was performed at the Institute of Rheumatology in Prague. Collection of clinical data and blood from patients as well as the laboratory analysis of the biological material was undertaken under the regulation of the relevant local research ethics committee (Ref. Nr. 5371/2012).

## Results and discussion

**Figure 1.**
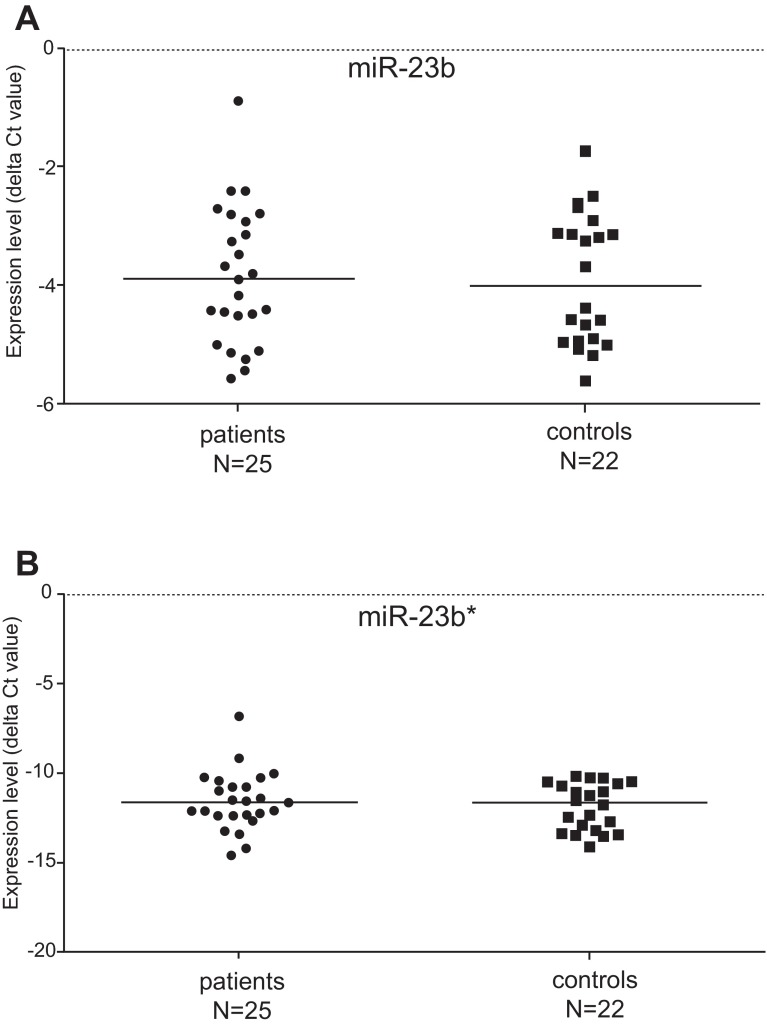
Regulation of the miR-23b and miR-23b* expression in patients and controls. The expression level of miR-23b was not significantly changed in patients suffering from idiopathic inflammatory myopathy when compared with healthy controls ( **A**). No significant difference in expression of miR-23b* was found between myositis patients and controls ( **B**).

We have examined the differential expression of the miR-23b and miR-23b* in PBMCs of patients with PM/DM and healthy controls. The detection of expression of miR-23b and miR-23b* was performed by quantitative real-time PCR. The median expression level (delta Ct) of miR-23b in the group of IIM patients was -3.90, the expression (delta Ct) in the control group reached -4.03. The median expression level of miR-23b* was -11.69 in IIM patients and -11.68 in the controls ( Figure 1). The expression level (delta Ct) of both tested miR-23b molecules did not show any significant differences between patients suffering from myositis and control cohort ( Figure 1). Furthermore, we did not find any difference in miR-23b (or miR-23b*) expression between DM and PM patients. The table with raw qPCR expression data used for statistical evaluation is provided in supplementary material.

Expression profiles of miR-23b and miR-23b in peripheral blood mononuclear cells of patients with dermatomyositis and polymyositis RNA was extracted from peripheral blood mononuclear cells (PBMCs) of patients and controls and TaqMan gene specific miRNA assays were used to quantify the expression levels of mature miR-23b and miR-23b* (miR23b star) molecules against controls (RNU44, RNU48, RNU6B, U47).

The role of miR-23b has been studied on inflammatory lesions of humans with lupus erythematosus or rheumatoid arthritis, as well as on corresponding tissues of murine models of lupus, rheumatoid arthritis and multiple sclerosis ^12^. The authors Zhu *et al.* ^12^ demonstrated that miR-23b targets TAB2, TAB3 and IKK-α mRNA transcripts and thereby suppresses autoimmune symptomatology by limiting the actions of the key proinflammatory cytokines IL-17, IL-1β and TNF. As it was found that the *in vitro* treatment of resident cell lines with IL-17 led to the downregulation of miR-23b, the expression of miR-23b and IL-17 in tissue resident cells seems to be mutually antagonistic ^12^. IL-17 is mainly produced by activated T-cells, has the ability to activate other T-cells, and is also capable of triggering the inflammatory process in many other tissues/cells ^16, 17^. Involvement of miR-23b in the pathogenesis of myositic muscle was recently reported ^12^ and the etiopathogenetic role of IL-17 in IIM has also been previously described ^18, 19^. The aim of our research was to analyze the changes in expression of miR-23b/miR-23b* in PBMCs from patients with myositis and to evaluate the importance of the IL-17/miR-23b signaling network in the PMBCs of these patients. Our results clearly show that the expression profile of miR-23b (as well as miR-23b*) is not changed in the PBMCs of IIM patients. Therefore, the regulative role of miR-23b in the development of myositis is not taking place in PMBCs. We cannot exclude involvement of this system in the local inflammatory process within the inflamed myositis muscle tissue.
