# Supplementary material for: The expression profile of miR-23b is not altered in peripheral blood mononuclear cells of patients with idiopathic inflammatory myopathies
Source: F1000Res. 2013 Oct 23;2:223. [Version 1] doi: 10.12688/f1000research.2-223.v1 (PMC3886791; doi:10.12688/f1000research.2-223.v1)
Supplement: Peer review history

# Peer review: Referee response for version 1

**Published:** 2013-12-11

**Authors:** Stefano Casola

**Affiliations:** 1 Genetics and Epigenetics of Lymphocyte Development and Transformation Research Program, Institute of Molecular Oncology Foundation (IFOM Institute), Milan, Italy

In this article the authors provide convincing evidence that expression of miRNas mir23 and mir23b* in peripheral blood mononuclear cells of patients affected by inflammatory myopathies is unchanged. This result warrants future investigation to elucidate the role of the same miRNAs in cells that take an active part in the inflammatory response in affected muscles.

Minor comment: The authors should spend one sentence to describe the existence of two separate mir23b miRNAs. Also, in the main text the authors should correct the acronym PBMC in a few places towards the end of the discussion.

I have read this submission. I believe that I have an appropriate level of expertise to confirm that it is of an acceptable scientific standard.

# Peer review: Referee response for version 1

**Published:** 2013-11-11

**Authors:** Maria Rescigno

**Affiliations:** 1 Department of Experimental Oncology, European Institute of Oncology, Milan, Italy

Overall I approve of this article; I would just add in the discussion that one caveat of this work is the use of PBMC and that by taking resident inflammatory cells the authors may be able to see a difference in miR-23 expression.

I have read this submission. I believe that I have an appropriate level of expertise to confirm that it is of an acceptable scientific standard.
